# Enhancing speaker identification through reverberation modeling and cancelable techniques using ANNs

**DOI:** 10.1371/journal.pone.0294235

**Published:** 2024-02-14

**Authors:** Emad S. Hassan, Badawi Neyazi, H. S. Seddeq, Adel Zaghloul Mahmoud, Ahmed S. Oshaba, Atef El-Emary, Fathi E. Abd El‑Samie

**Affiliations:** 1 Department of Electrical Engineering, College of Engineering, Jazan University, Jizan, Saudi Arabia; 2 Department of Electronics and Electrical Communications Engineering, Faculty of Electronic Engineering, Menoufia University, Menouf, Egypt; 3 Productivity and Vocational Training Department, Ministry of Industry, Cairo, Egypt; 4 Acoustic Laboratory, Housing and Building National Research Center, Giza, Egypt; 5 Electronics and Communications Department, Faculty of Engineering, Zagazig University, Zagazig, Egypt; 6 College of Engineering, Deltal University for Science and Technology, Mansoura, Egypt; 7 Department of Information Technology, College of Computer and Information Sciences, Princess Nourah Bint Abdulrahman University, Riyadh, Saudi Arabia; Institute of Theoretical and Applied Informatics Polish Academy of Sciences: Instytut Informatyki Teoretycznej i Stosowanej Polskiej Akademii Nauk, UKRAINE

## Abstract

This paper introduces a method aiming at enhancing the efficacy of speaker identification systems within challenging acoustic environments characterized by noise and reverberation. The methodology encompasses the utilization of diverse feature extraction techniques, including Mel-Frequency Cepstral Coefficients (MFCCs) and discrete transforms, such as Discrete Cosine Transform (DCT), Discrete Sine Transform (DST), and Discrete Wavelet Transform (DWT). Additionally, an Artificial Neural Network (ANN) serves as the classifier for this method. Reverberation is modeled using varying-length comb filters, and its impact on pitch frequency estimation is explored via the Auto Correlation Function (ACF). This paper also contributes to the field of cancelable speaker identification in both open and reverberation environments. The proposed method depends on comb filtering at the feature level, deliberately distorting MFCCs. This distortion, incorporated within a cancelable framework, serves to obscure speaker identities, rendering the system resilient to potential intruders. Three systems are presented in this work; a reverberation-affected speaker identification system, a system depending on cancelable features through comb filtering, and a novel cancelable speaker identification system within reverbration environments. The findings revealed that, in both scenarios with and without reverberation effects, the DWT-based features exhibited superior performance within the speaker identification system. Conversely, within the cancelable speaker identification system, the DCT-based features represent the top-performing choice.

## 1. Introduction

The analysis of speech signals serves as a powerful tool for individual characterization, encompassing aspects such as identity, dialect, age, emotional state, language, gender, and even health status. Each person possesses distinct natural vocal characteristics that distinguish him. Speech has been a fundamental mode of human communication since ancient times, arising from vocal tract excitation. Physiological attributes contributing to speech differ across individuals, including variations in vocal tract size, shape, vocal fold structure, velum, and nasal cavity, especially between genders [1–3].

Speaker recognition is a signal processing technique that aims to identify individuals based on their spoken words. It encompasses two primary categories: Speaker Identification (SI) and Speaker Verification (SV). Identification involves comparing an enrolled voice with stored models to identify the best match, while verification confirms or rejects a claimed identity. SV finds applications in security contexts. Both SI and SV involve the creation of speaker models to be stored as references [4, 5]. The process of SV is also referred to as speaker authentication, wherein the system either accepts or rejects the speaker’s identity claim. If the system denies access to an enrolled speaker’s utterance, the speaker is classified as an impostor. Consequently, SV systems play a crucial role in security applications, thwarting unauthorized entry by individuals [6, 7].

Speaker identification involves recognizing the speakers’ identities by comparing their feature vectors with those stored in the database. For unknown speakers, the system matches their voice models with the existing database, assigning the best-fitting model as the unknown speaker’s representation. This application extends to domestic domains like forensics and identification of individuals involved in criminal cases within a pool of known offenders [2].

Automatic Speaker Identification (ASI) comprises two stages: feature extraction and classification. Feature extraction condenses speech signals into concise data, forming feature vectors that encapsulate distinct speaker characteristics. The speaker identification system operates in training and recognition modes. During training, features of new speakers are extracted and recorded in the database, while recognition involves extracting features for unknown speakers to determine their identities. Mel-Frequency Cepstral Coefficients (MFCCs), widely acclaimed for their robustness in representing clean speech, are the favored features [3, 6]. However, their robustness diminishes in cases of degraded speech quality.

This paper extensively investigates the impact of closed-room environments on speech signals. This impact arises from the numerous reflections occurring off the walls within such spaces. In specific settings, substantial reverberation is anticipated [8, 9]. Consequently, it is likely that the features extracted from speech signals exhibit variances in the presence of reverberation. The exploration extends to the degree of influence exerted by reverberation on cepstral features and pitch frequency, as well as its impact on the whole speaker identification process.

Over the past decade, the notion of cancelable biometrics has undergone significant development. This concept holds particular relevance for enhancing the security of biometric systems, especially those utilized in remote-access scenarios. Cancelable biometrics relies on the utilization of distorted signals or feature patterns, which are extracted to represent speakers [10]. In this paper, the concept of a cancelable speaker identification is adopted by employing a digital comb filter, analogous to the model used for simulating reverberation. It is well-established that reverberation can be effectively modeled using a comb filter. Therefore, an additional comb filter is implemented at the feature level to induce deformations within the features. Subsequently, the impact of these deformations on the speaker identification process is analyzed.

In summary, this paper advances the fields of speaker identification and cancelable biometrics, offering effective solutions for challenging acoustic conditions. The key contributions of this paper can be summarized into the following points:

1. Reverberation analysis and modeling: The paper explores the analysis of speech signals in environments with reverberation caused by reflections from closed room surfaces. The reverberation is modeled using comb filters with varying lengths, offering a methodical approach to simulating and understanding its effects.
2. Robust speaker identification: The paper presents a robust speaker identification system designed to operate effectively in scenarios with both reverberation and noise, leveraging MFCCs.
3. Cancelable speaker identification: Addressing contemporary trends in biometric security, the paper introduces cancelable speaker identification for both open and reverbration environments. A novel technique involves applying comb filtering at the feature level, distorting MFCCs to obscure speaker identities and enhance security.
4. ANN classification: The proposed cancelable speaker identification system employs ANNs for classification, achieving high recognition rates in the cancelable biometric recognition framework.
5. Finally, the paper outlines three distinct systems: a reverberation-affected speaker identification system, a system depending on cancelable features obtained through comb filtering, and a novel cancelable speaker identification system tailored for challenging reverberation environments.

## 2. Related work

The study of speech signals in reverbration environments and the development of robust speaker identification systems have garnered significant attention in recent years. This section presents an overview of relevant research in the areas of speech signal analysis, speaker identification, and cancelable biometrics.

Understanding the effects of reverberation on speech signals is a critical aspect. Prior works have investigated various aspects of reverberation modeling and its impact on speech features. Dealing with reverberation in speech processing has been addressed through techniques like dereverberation, which aims to mitigate the adverse effects of reverberation on speaker recognition systems [11]. Methods, such as adaptive filtering and beamforming, have been employed to enhance the quality of reverberant speech [12].

Furthermore, studies have explored the modeling of reverberation using comb filtering, which is utilized to simulate room acoustics and evaluate the performance of speech processing algorithms in reverbration conditions [10]. Traditional speaker identification systems rely on extracting features from speech signals and matching them with reference models [4]. MFCCs have been a common choice for feature extraction due to their effectiveness in clean speech conditions. However, their robustness in the presence of reverberation and noise is a subject of ongoing investigation [3, 6].

Cancelable biometrics has emerged as a promising approach to enhance security in biometric systems. The concept of cancelable biometrics involves the deliberate distortion of biometric features to generate cancelable templates, ensuring that the original biometric data remains protected [10]. Research in this domain has explored various methods for generating cancelable templates, including the introduction of controlled noise, feature-level transformations, and comb filtering. Cancelable biometrics offers potential solutions to privacy concerns and security threats in biometric authentication systems.

Artificial Neural Networks (ANNs) have demonstrated remarkable capabilities in extracting intricate patterns from speech features, enabling high-accuracy speaker recognition systems [13]. The utilization of deep learning architectures, such as Convolutional Neural Networks (CNNs), has further improved the performance of speaker identification models [13]. These developments highlight the potential for ANNs to play a pivotal role in cancelable speaker identification systems. Challenges posed by reverbration environments have been addressed in the literature, with researchers proposing various strategies to enhance speaker identification performance in such conditions. These strategies include the adaptation of feature extraction methods to account for reverberation effects, the utilization of multi-microphone arrays for source separation and dereverberation, and the incorporation of robust feature selection techniques [14, 15].

The authors of [16] developed a semi-sequential two-stage system that combines generative Gaussian Mixture Model (GMM) and discriminative Support Vector Machine (SVM) classifiers with prosodic and short-term spectral features for concurrent gender and identity classification. It operates in a two-stage, semi-sequential manner. The first classifier employs prosodic features to ascertain the speaker’s gender, which is then integrated with short-term spectral features as inputs into the second classifier that is used for speaker identification. This second classifier depends on two types of short-term spectral features, specifically MFCCs and Gammatone Frequency Cepstral Coefficients (GFCCs), in addition to gender information, resulting in the creation of distinct classifiers. The outputs from the different types of second-stage classifier, namely GMM-MFCC Maximum Likelihood Classifier (GMM-GFCC MLC), and GMM-GFCC supervector SVM, are amalgamated at the score level through the weighted Borda count approach. However, none of these prior works explored the use of discrete transforms for feature extraction in the context of speaker identification and cancelable speaker identification systems. Therefore, in this study, we address this gap by investigating the incorporation of discrete transforms into the feature extraction process. Additionally, this paper introduces a novel contribution by applying comb filtering to introduce distortion to MFCCs at the feature level. This distortion is integrated into a cancelable biometric framework, enhancing the system ability to conceal speaker identities and bolstering its resistance to potential intruders.

## 3. Speaker identification process

**Fig 1:**
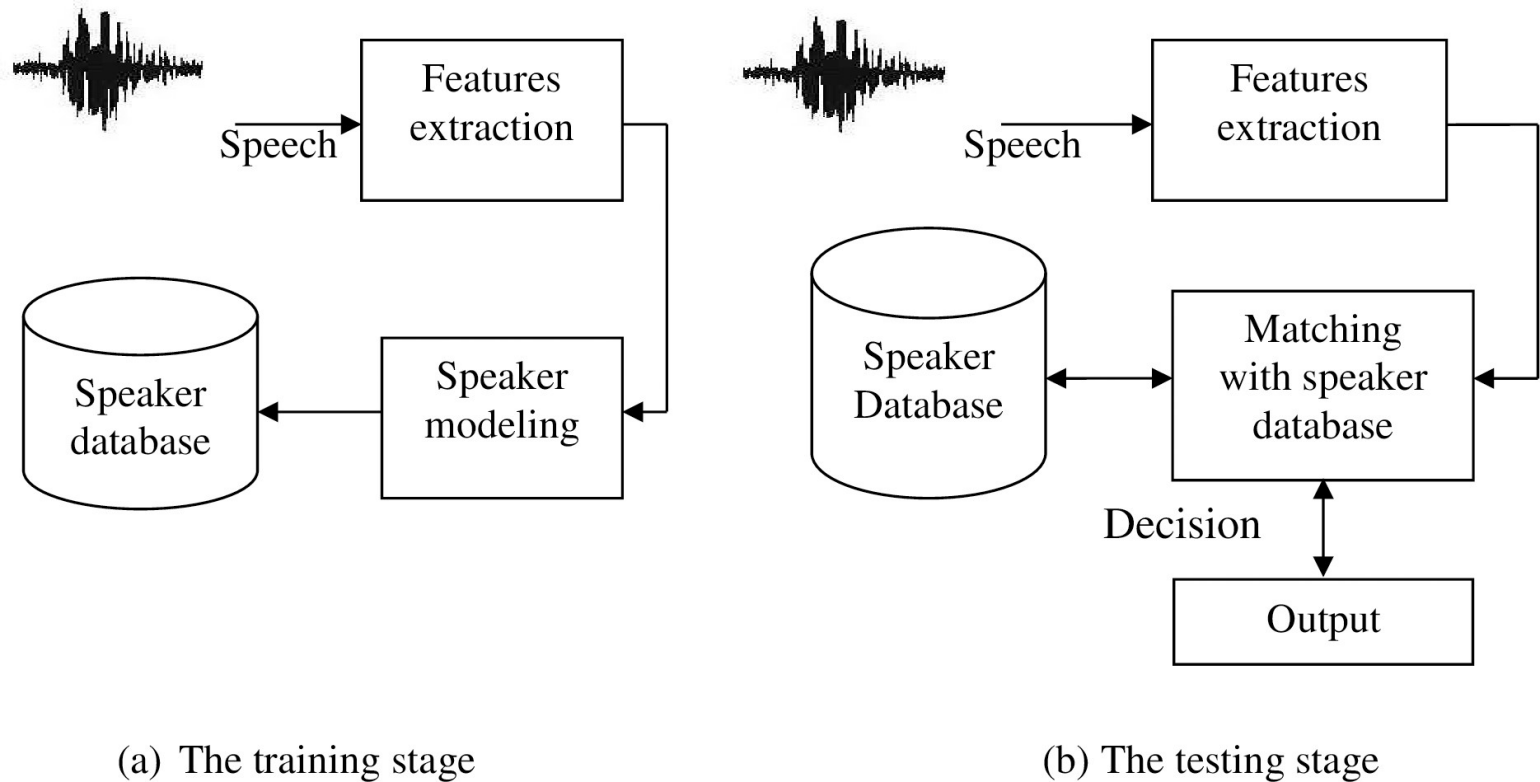
Training and testing stages of an ASI system.

The term "feature extraction" is often synonymous with the initial phase of speaker identification. This process plays a pivotal role in both the training and testing phases, as depicted in Fig 1. Serving as the cornerstone, feature extraction captures the paramount information for Automatic Speaker Identification (ASI). It effectively eliminates redundancy, while transforming the speech signal into a suitable format compatible with the classification model. This is achieved by discerning a series of attributes within the speaker’s utterance, referred to as features, which encapsulate the distinctive traits of each utterance. These features harbor discriminative properties tailored to individual utterances, encapsulating their intrinsic characteristics. Regarded as a data reduction step, feature extraction condenses lengthy utterances into compact data that encapsulates the core attributes of the speaker [1].

In summary, feature extraction is unequivocally the linchpin driving the success of the ASI system. Various factors can influence this process, including human-related aspects like inaccuracies in prompted phrase reading, and environmental variables like disparities in recording channels indicating the use of distinct microphones for training and testing as well as recordings conducted in noisy surroundings. Additionally, classification stands out as a pivotal phase within any speaker identification system [14–17].

The classification procedure comprises two distinct phases: training and testing. During the training phase, the extraction of distinctive features from speech samples belonging to registered speakers is imperative. This culminates in the creation of a unique pattern for each speaker that is subsequently archived in a database for later deployment in the matching process. Subsequently, in the testing or matching stage, upon the entry of an unidentified speaker into the system, features are extracted from his speech signal, and correlation is estimated between the models stored in the database and the model derived from the unknown speaker’s utterance. Based on the resulting matching score, a decision is rendered, gauging the similarity between the unknown speaker’s model and the database models. Ultimately, the model that best aligns with the unknown speaker’s model is designated as the speaker’s representative model.

## 4. Feature extraction stages

**Fig 2:**
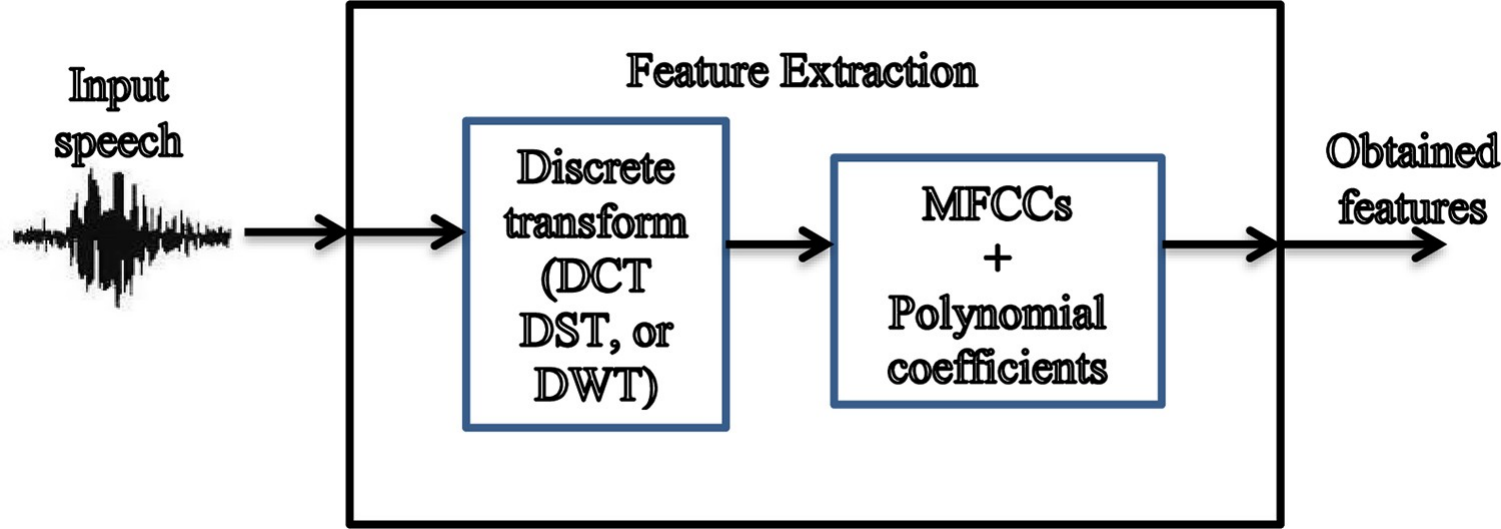
Features from different transforms.

The feature extraction has some stages for robust human auditory system representation. Some transformations are used to extract the most important information, as shown in Fig 2.

### 4.1 Utilization of discrete transforms

In the realm of speaker identification systems, discrete transform domains can give more representative MFCCs. This section delves into the exploration of three pivotal discrete transforms; the Discrete Cosine Transform (DCT), the Discrete Sine Transform (DST), and the Discrete Wavelet Transform (DWT) [18–21]. All of which hold potential for robust MFCC extraction. The forthcoming sub-sections will introduce these transformation techniques and elucidate their outcomes within the scope of the ASI system.

#### 4.1.1 Discrete Cosine Transform (DCT)

The DCT, akin to a Fourier-related transform, exclusively operates with real numbers. Its computation mirrors that of the Discrete Fourier Transform (DFT) conducted on a dataset nearly twice its length. This transform specifically suits real-valued data with even symmetry and exhibits an intriguing energy compaction trait. The significance of this property lies in the potential concentration of speech signal energy into few coefficients. In scenarios where the bulk of energy is channeled into a limited number of coefficients, a succinct set of features would aptly capture the distinct attributes of speakers [18, 19].

$$X(k)=\alpha (k)\sum_{n=0}^{N-1}x(n)cos(\frac{\pi (2n+1)k}{2N}),\;k=0,1,2,\ldots ,N-1$$ (1)

where *N* is the number of subcarriers, 0≤*n*≤*N*−1, and $\alpha (0)=\sqrt{\frac{1}{N},}\;\alpha (k)=\sqrt{\frac{2}{N}}$

The Inverse DCT (IDCT) is expressed as:

$$x(n)=\sum_{n=0}^{N-1}\alpha (k)X(k)cos(\frac{\pi (2n+1)}{2N}),\;n=0,1,2,\ldots ,N-1$$ (2)

#### 4.1.2 Discrete Sine Transform (DST)

The DST similarly aligns with the Fourier-related transform category. Corresponding to the imaginary component of the DFT conducted on a dataset nearly twice its length, the DST operates on real data, and it is distinguished by odd symmetry. This choice stems from the principle that the Fourier transform of a real and odd function results in an imaginary and odd function. Variants of the DST might also involve shifting input and/or output data by half a sample. Mathematically, for a given sequence *x*(*n*), the DST is defined as [20]:

$$x(k)=\sum_{n=0}^{N-1}x(n)sin(\frac{\pi }{N+1}(n-1)(k+1)),k=0,1,\ldots ,N-1$$ (3)

#### 4.1.3 Discrete Wavelet Transform (DWT)

**Fig 3:**
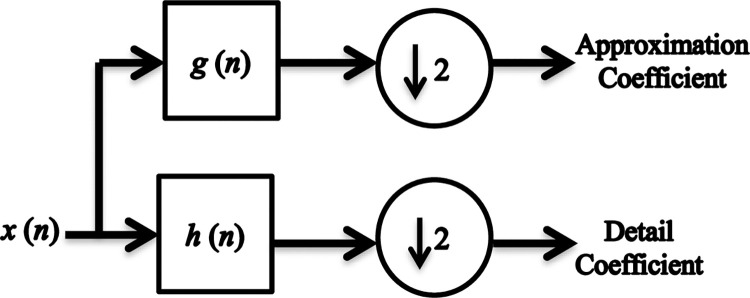
Wavelet transform.

Wavelet transform, as a mathematical procedure, facilitates the partitioning of an audio signal into different sub-bands of varying scales, enabling the independent study of each scale. The DWT is built on the principle of segregating a signal into two key components of low-frequency (approximation) and high-frequency (details) natures, respectively. This involves subjecting the speech signal to a low-pass filter yielding the approximation signal, and a high-pass filter producing the detail signal. Both of these resulting signals hold potential for modeling the characteristics of the speech signal. A graphical depiction of the wavelet transform is given in Fig 3 [21, 22].

The outputs of the two filters can be expressed as:

$$y_{low}(n)=\sum_{k=-\infty }^{\infty }x(k)h(2n-k)$$ (4)

$$y_{high(n)}=\sum_{k=-\infty }^{\infty }x(k)g(2n-k)$$ (5)

### 4.2 MFCCs

Human speech encapsulates a plethora of speaker-specific attributes, highly valued as discriminative attributes that can be exploited in the recognition process. Among the most prominent low-level features, MFCCs stand out. The generation of speech is characterized by a filter model that represents the vocal tract through its impulse response *h*(*n*) and an input source *e*(*n*). This process is illustrated in Eq (6),

s(n)=e(n)*h(n) (6)

where *s*(*n*) signifies the speech signal formed by convolving *e*(*n*) and *h*(*n*) within the temporal domain [23].

In the process of speech production, a substantial volume of data is generated. While a portion of this data embodies crucial speaker-specific attributes, a significant portion is deemed superfluous. The fundamental objective of feature extraction revolves around minimizing data size, while preserving solely the speaker-discriminative information. Within this context, the vocal tract is responsible for the spectral envelope, governing low spectral variations, whereas the excitation source governs spectral nuances, entailing high spectral variations [24]. In an ASI, the spectral envelope has a paramount significance over the details, as it holds the most distinguishing features. Consequently, the isolation of the spectral envelope from the details has a pivotal importance. This separation between the vocal tract and the excitation source is effectively accomplished through cepstrum evaluation [24].


Cepstrum(Frame)=IFFT(log(|FFT(Frame)|)) (7)

Taking FFT of Eq (6),

S(ω)=E(ω)H(ω) (8)

The logarithm maps the multiplication into addition as follows [24]:

log(S(ω))=log(E(ω))+log(H(ω)) (9)

By translating multiplication into addition, a seamless separation of *E*(*ω*) from *H*(*ω*) is facilitated, especially post IFFT application, where the operation is executed on individual terms. This action yields what is known as the cepstrum domain. In this domain, frequency maps to quefrency. *E*(*ω*), the excitation spectrum, corresponds to high spectral variations (details) predominantly found in high quefrency, while *H*(*ω*), the vocal tract, accounts for low spectral variations (envelope) present at low quefrency. Evidently, research has validated the information-rich nature of the speech spectrum envelope compared to its details [25].

**Fig 4:**
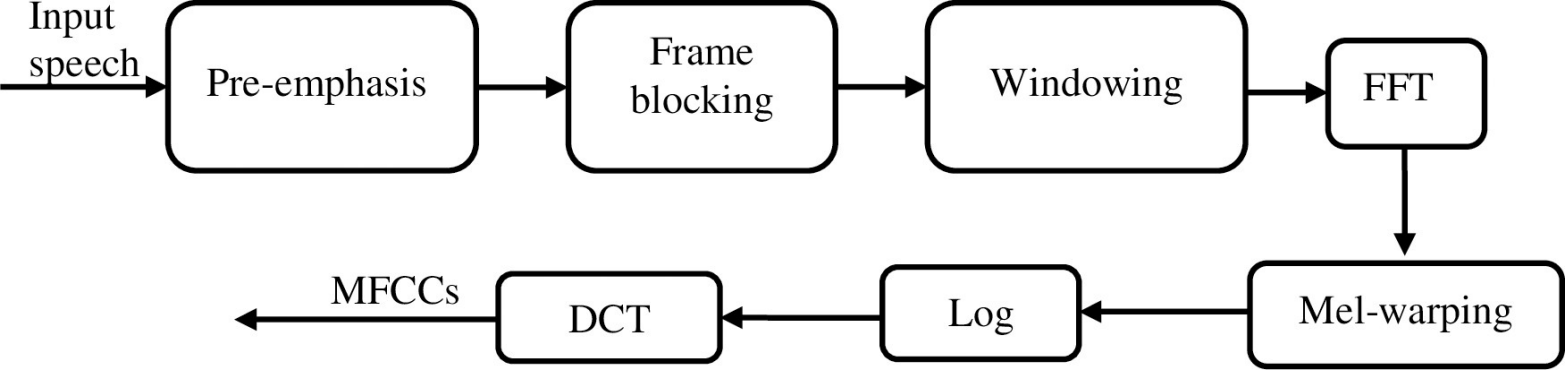
MFCCs extraction.

Within this context, MFCCs emerge as the preferred choice due to their superior alignment with the human auditory system response [25]. This alignment is achieved through the Mel-scale, which takes into consideration the frequency bands of the auditory system. Human auditory system does not perceive frequencies beyond 1 kHz linearly; instead, it adheres to a logarithmic scale above this threshold while maintaining linearity below. To bridge this, the MFCCs method employs two kinds of filters: linear-spaced filters below 1 kHz and logarithmic-spaced filters above 1 kHz [26–28]. Computation of MFCCs centers on short-term analysis, following a standardized procedure. It entails the initial framing and windowing of speech signals, followed by FFT computation. The resultant spectrum is then transformed into the Mel scale [27]. Subsequent steps involve applying the logarithm to the scaled spectrum and performing the DCT, as outlined in Fig 4.

### 4.3 Polynomial coefficients

The attained MFCCs, in themselves, prove insufficient for comprehensive information extraction. Thus, the integration of polynomial coefficients with them serves to bolster the system resilience against discrepancies encountered during the matching process. It is through these polynomial coefficients–encompassing attributes like curvature, mean, and slope–that the core insights are gleaned from the cepstral coefficients. Remarkably, the temporal profiles of specific cepstral coefficient sets consistently demonstrate analogous behaviors in both training and testing, despite variations in coefficient amplitudes across these stages. This underscores the constancy in the temporal forms of selected cepstral coefficients from training to testing [29].

Extending the cepstral coefficients’ scope involves employing orthogonal polynomial-based time waveform modeling, which, in turn, enables the calculation of polynomial coefficients. The embodiment of these orthogonal polynomials assumes the following mathematical expressions:

$$p_{1}(i)=i-5$$ (10)

$$p_{2}(i)=i^{2}-10\;i+\frac{55}{3}$$ (11)

The modeling of MFCCs is formed using a nine-element window for each MFCC. The polynomial coefficients are given by:

$$a_{j}(t)=\frac{\sum_{i=1}^{9}p_{1}(i)c_{j}(t+i+1)}{\sum_{i=1}^{9}{p_{1}}^{2}(i)}$$ (12)

$$b_{j}(t)=\frac{\sum_{i=1}^{9}p_{2}(i)c_{j}(t+i+1)}{\sum_{i=1}^{9}{p_{2}}^{2}(i)}$$ (13)

Here, *a*_*j*_(*t*) pertains to the slope, while *b*_*j*_(*t*) represents the curvature within the MFCCs time functions. The resultant feature vector encompasses *a*_*j*_(*t*), *b*_*j*_(*t*), and *c*_*j*_(*t*) representing the MFCCs.

Consciously, the extraction of features involves seven distinct methodologies encompassing:

1. Features sourced from the speech signals.
2. Features derived from the DWTs of the speech signals.
3. Features obtained from both the speech signals and their associated DWTs.
4. Features derived from the DCTs of the speech signals.
5. Features derived from both the speech signals and their associated DCTs.
6. Features originating from the DSTs of the speech signals.
7. Features obtained from both the speech signals and their associated DSTs.

This technique is embraced during the testing phase to emulate the performance of the human auditory system when handling degraded speech. The evaluation of the ASI system performance is gauged through recognition rates stemming from different signal transforms. The recognition rate is expressed as follows:

$$Recognition\;Rate\;(RR)=\frac{Number\;of\;success\;identifications}{Total\;number\;of\;identification\;trials}$$ (14)

Speaker-specific information contained within speech signals can be categorized into two distinct types: low-level information, delineated by the anatomical structure of the vocal tract; and high-level information, defined by learned behavioral habits and styles. Remarkably, the human brain possesses the capacity to distinguish individuals based on these high-level attributes, encompassing prosody, linguistic nuances, phonetic distinctions, emotional cues, language preferences, dialect, and lexical choices. When encountering an unfamiliar voice, a human can often identify the speaker by analyzing these attributes.

In contrast, the ASI system, a machine learning entity, processes speech information using low-level features rooted in physical traits like the larynx and vocal tract. These features represent distinct speech and speaker-dependent vocal tract configurations. Given that variations in the shape and size of the vocal tract and laryngeal tract result in speaker-specific information embedded in the speech signals, constructing a speaker identification system founded solely on behavioral traits becomes unfeasible. Hence, an ASI system founded upon low-level features stands as a more practical tool.

## 5. Classification process

The process of identification unfolds in a two-fold manner: encompassing speaker training (modeling) and speaker matching stages. During the training or modeling phase, an individual model is constructed for each speaker based on features extracted from his spoken utterances, and subsequently stored within a database. In the subsequent matching stage, when an unidentified speaker provides utterances, akin features to those garnered during training are extracted from the provided speech segment. Subsequently, the generated model is juxtaposed against models housed within the database, facilitating the identification of the best-matched model for the unknown speaker, thereby informing the ultimate decision.

Different classifiers can be used in this identification process, including Gaussian Mixture Models (GMMs), Hidden Markov Models (HMMs), Vector Quantization (VQ), Support Vector Machines (SVMs), and Artificial Neural Networks (ANNs). Within this context, the employment of ANNs is prominent [30, 31].

### 5.1 Artificial Neural Network (ANN) classifier

ANNs serve as simulation models for the human brain functions, emulating the brain capacity to perform complex tasks by processing data in a manner akin to human cognition [29, 30]. Structured with an assembly of numerous simple processing units known as neurons, ANNs are interlinked through connections denoted as weights. This arrangement follows an organizational framework comprising an input layer, potentially multiple hidden layers, and an output layer. Each layer is composed of cells, with these cells interconnected by weights that facilitate the flow of information from input through hidden layers to the output layer.

Training ANNs hinges on weight adjustments between neurons. The learning process can take the form of supervised learning, in which the network is presented with an input and the corresponding desired output. Alternatively, unsupervised learning, also termed self-organized learning, necessitates input alone, prompting the network to independently adapt based on the input data. Reinforcement learning is yet another approach where the network fine-tunes its weights in response to input data until the accurate output is achieved.

### 5.2 ANN computations

Upon introducing the input pattern to the input neurons, the activations of all neurons are computed. The learning process involves adjusting the weight strengths until the network effectively learns to compute a specific function mapping input to output, or autonomously classify input data. This unidirectional flow from input to output is known as feed-forward propagation, with the network devoid of feedback. Conversely, in feedback propagation networks, output-to-input feedback is present.

**Fig 5:**
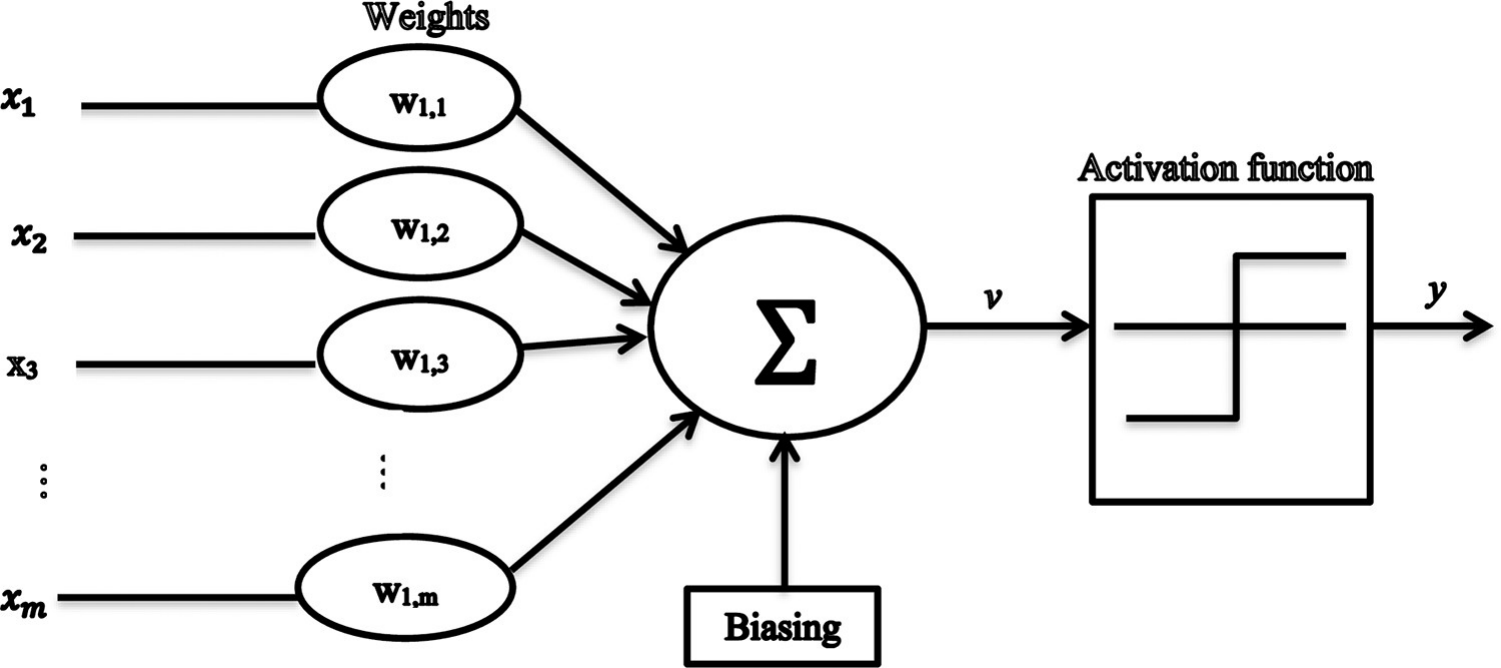
Computation of the net activation.

Each neuron update follows a two-step process: first, computation of the net input for the neuron is executed; subsequently, the activation output is calculated based on this net input. If we denote an *m*-element vector as **x** = [*x*_1_, *x*_2_, *x*_3_, *…*., *x*_*m*_], it serves as the input to the neuron. Through multiplication by weights *w*_11_, *w*_12_, *w*_13_, *…*.., *w*_1*m*_, the net input to the activation function *v* is generated, as depicted in Fig 5 [31].


$$u_{k}=\sum_{i=1}^{m}x_{i}w_{ji}$$ (15)

$$v_{k}=u_{k}+b_{k}$$ (16)

$$y_{k}=f(v_{k})$$ (17)

Here, *x*_*i*_ denotes input data, *b*_*k*_ represents the bias, and *w*_*ji*_ signifies the weight originating from unit *i* to *j*. Subsequently, the net input is employed as the argument for the activation function. Upon computing the net input, the activation output is determined through a function dependent on *v*_*j*_. Additionally, within this context, *f* denotes the activation function, *y* stands for the neuron output, and *b* serves as the bias contributing to the refined transformation of the output *v*_*j*_.

## 6. Speech quality measurements

The clarity of speech hinges on the quality of both hearing and comprehending the spoken words, encompassing the accurate perception of verbal content. In numerous speech processing contexts, enhancing speech quality involves gauging the improvement in a specific portion of speech. This assessment is facilitated through speech quality metrics that fall into two primary categories: subjective and objective evaluations.

Subjective quality metrics are rooted in the perspective of listeners, who engage in a comparison between the original speech and the processed version. Consequently, speech quality is ascertained based on listeners’ perception, and a comprehensive evaluation emerges from the aggregation of results across multiple listeners. Contrarily, objective speech quality metrics depend on quantifiable measurements.

Objective metrics for speech quality are deduced from both the unaltered and impaired speech signals, employing mathematical formulations. These metrics offer efficiency and expedience, given their independence of listener involvement. Noteworthy objective speech quality metrics encompass Signal-to-Noise Ratio (SNR) and segmental Signal-to-Noise Ratio (SNRseg) [32].

### 6.1 Signal-to-Noise Ratio

The SNR, which stands as the oldest and extensively employed objective metric, is characterized by the following equation:

$$SNR=10{log}_{10}\frac{\sum_{i=1}^{N}x^{2}(i)}{\sum_{i=1}^{N}{\left(x(i)-y(i)\right)}^{2}}$$ (18)

In this equation, *x*(*i*) denotes the original speech, *y*(*i*) represents the impaired speech, and *i* corresponds to the sample index. Calculating the SNR involves straightforward mathematical steps, yet it necessitates access to both pristine and corrupted speech samples.

### 6.2 Segmental SNR

The SNR_seg_ gives the SNR over short frames, and then the average is estimated.


$${SNR}_{seg}=\frac{10}{M}\sum_{m=0}^{M-1}{log}_{10}\sum_{i=Nm}^{Nm+N-1}{\left(\frac{x^{2}(i)}{x(i)-y(i)}\right)}^{2}$$ (19)

In this context, where *N* signifies the frame length, typically falling within the range of 15 to 20 ms, and *M* denotes the count of frames within the speech signal, *x*(*i*) pertains to the initial speech, and *y*(*i*) stands for the altered speech [33].

## 7. Proposed systems

Three systems are presented in this section: a reverberation-affected speaker identification system, a system depending on cancelable features obtained through comb filtering, and a novel cancelable speaker identification system within reverberation environments.

### 7.1 Conventional speaker identification

**Fig 6:**
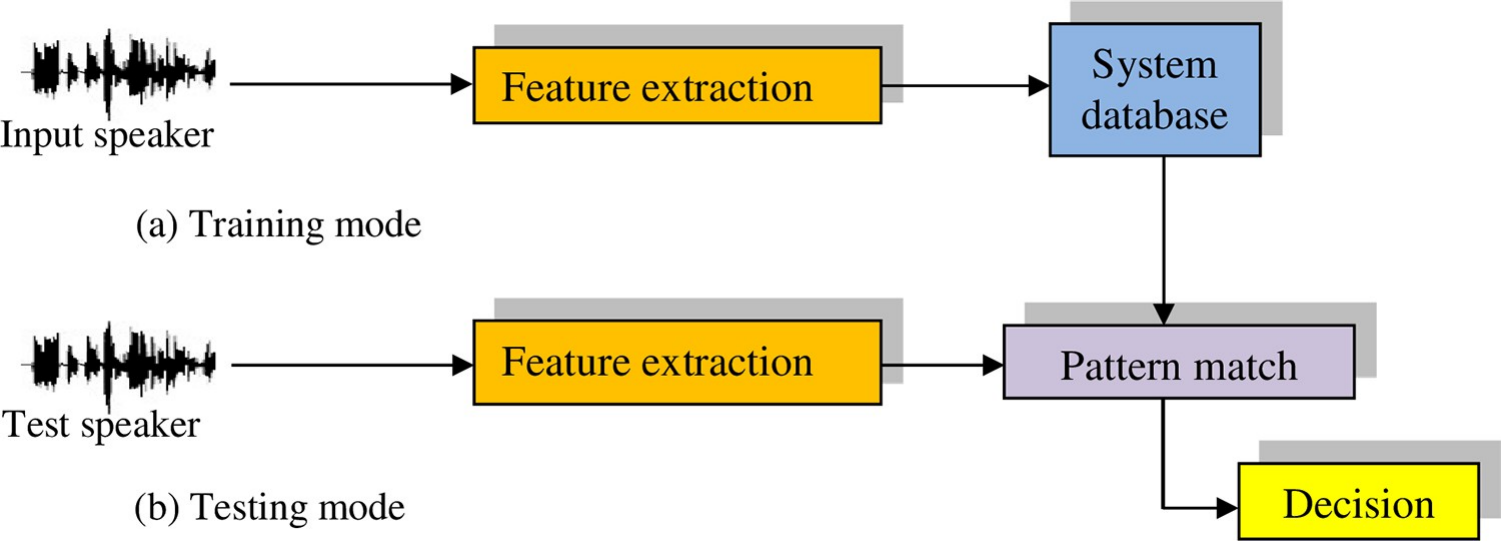
Conventional speaker identification system.

In this sub-section, the conventional speaker identification system is presented as a benchmark, in which the following steps are performed as shown in the Fig 6.

1. Feature extraction from the voice signals for training. Then, the model created using the neural network is saved in the database (Training mode).
2. Feature extraction from the unknown speaker voice signal. Then, matching with all speaker models in the database is performed for identification (Testing mode).

### 7.2 Proposed speaker identification system in the presence of reverberation

**Fig 7:**
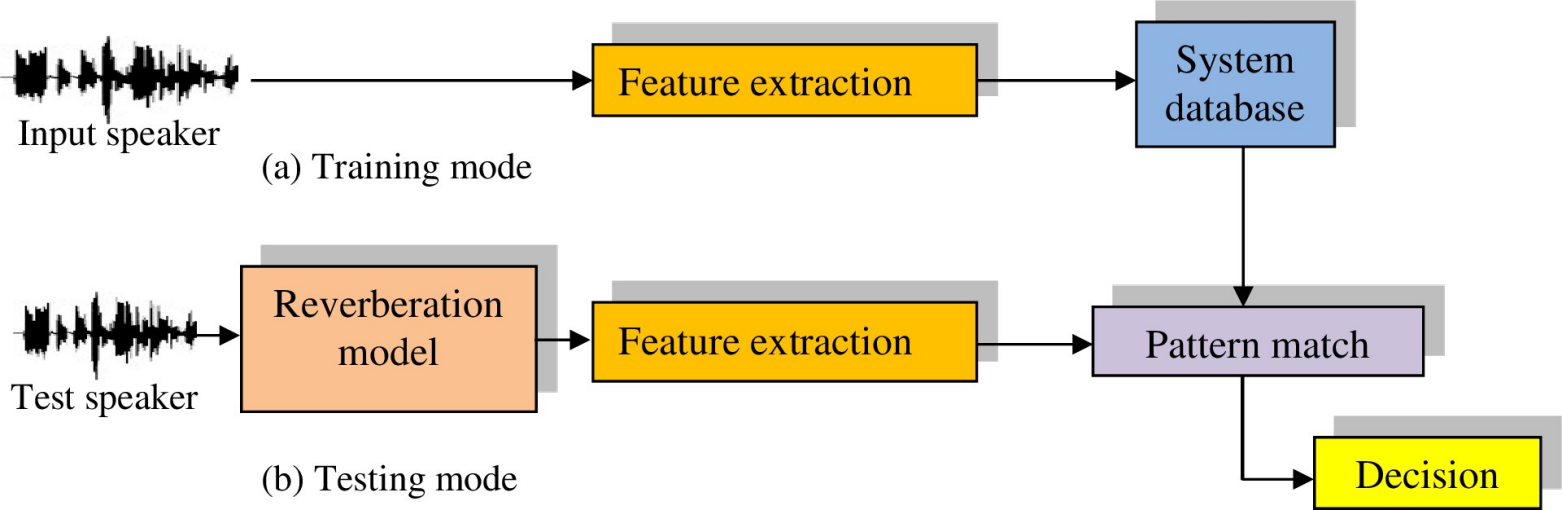
Proposed speaker identification in the presence of reverberation.

In this sub-section, we a present a speaker identification system in the presence of reverberation, in which the following steps are performed as shown in the Fig 7.

1. Feature extraction from the voice signals for training. Then, the models created using the neural network are saved in the database (Training mode).
2. Feature extraction from the reverberant speech signals (unknown speaker voices passed through comb filter), and then matching is performed with all speaker models in the database for identification, and then the decision is made (Testing mode).

#### 7.2.1 Reverberation modeling

The reverberation can be modeled with a comb filter that is applied on the original speech signal. It is, in fact, a multi-band filter represented as [8]:

$$H(z)=1-\frac{1}{Z^{L}}=\frac{Z^{L}-1}{Z^{L}}$$ (20)

**Fig 8:**
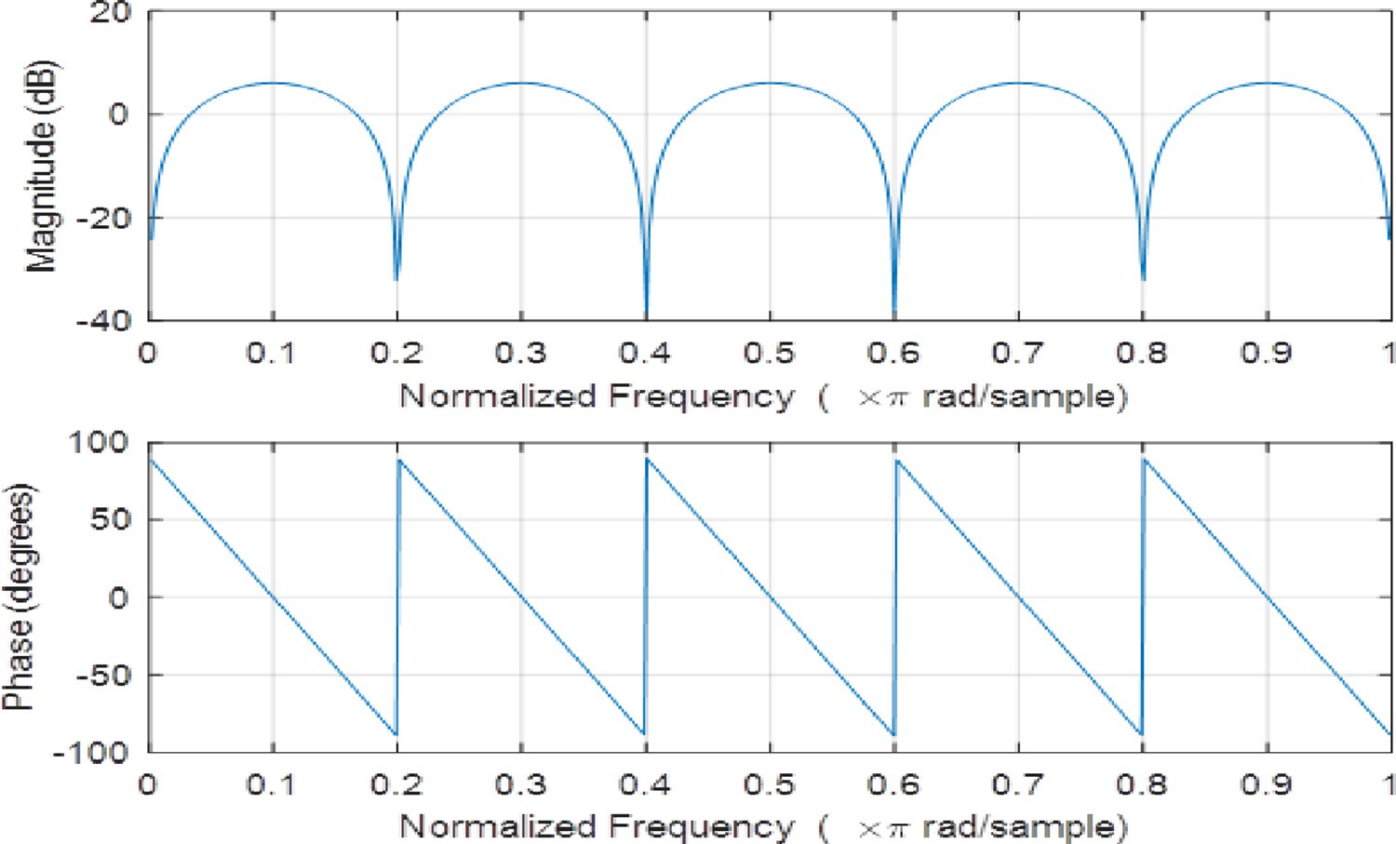
Magnitude and phase responses of a comb filter.

**Fig 9:**
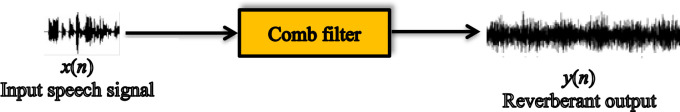
Comb filter and its output.

The discrete-time representation of this equation is given by:

y(n)=x(n)−x(n−L) (21)

where *L* is the filter length, which is proportional to the reverberation time. Both magnitude and phase responses of the comb filter of order 8 are given in Fig 8.

y(n)=x(n)*h(n) (22)

where *x*(*n*) refers to the input speech signal, *h*(*n*) indicates impulse response of the comb filter shown in Fig 9, and *y*(*n*) is the reverberant output.

### 7.3 Proposed cancelable speaker identification system

**Fig 10:**
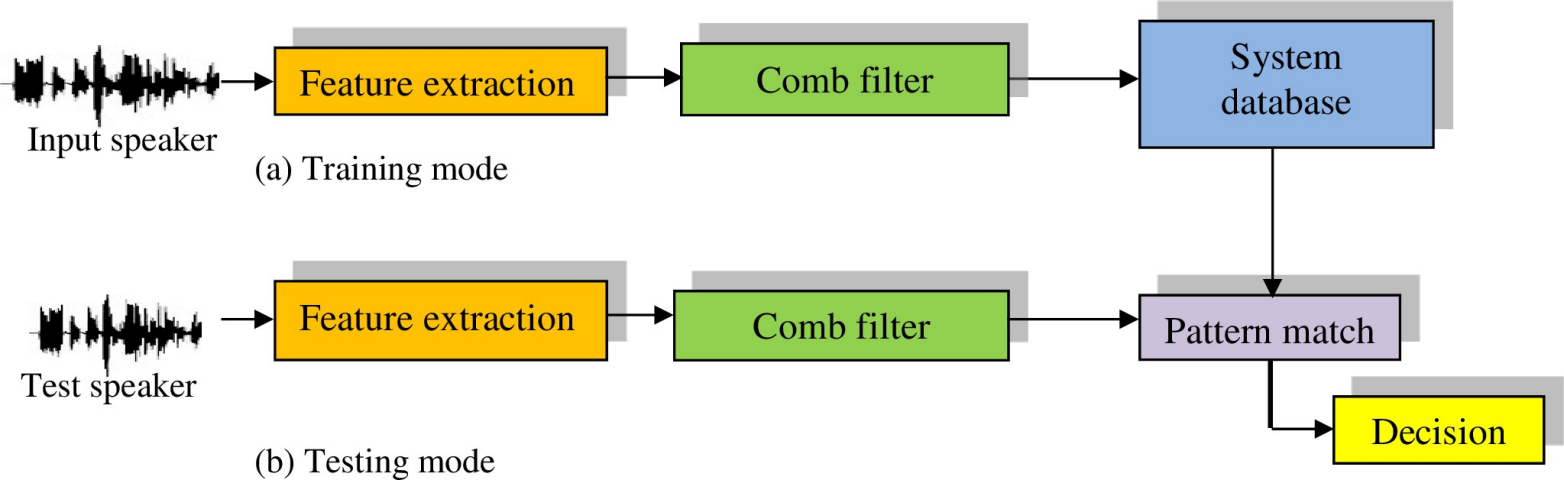
Proposed cancelable speaker system.

In this sub-section, we present a speaker identification system using cancelable features with comb filter as a distortion tool. In this case, both training and testing are performed with the comb filter effect as a tool for inducing distorsion as shown in the Fig 10.

### 7.4 Proposed cancelable speaker identification system on the feature level in the presence of reverberation

**Fig 11:**
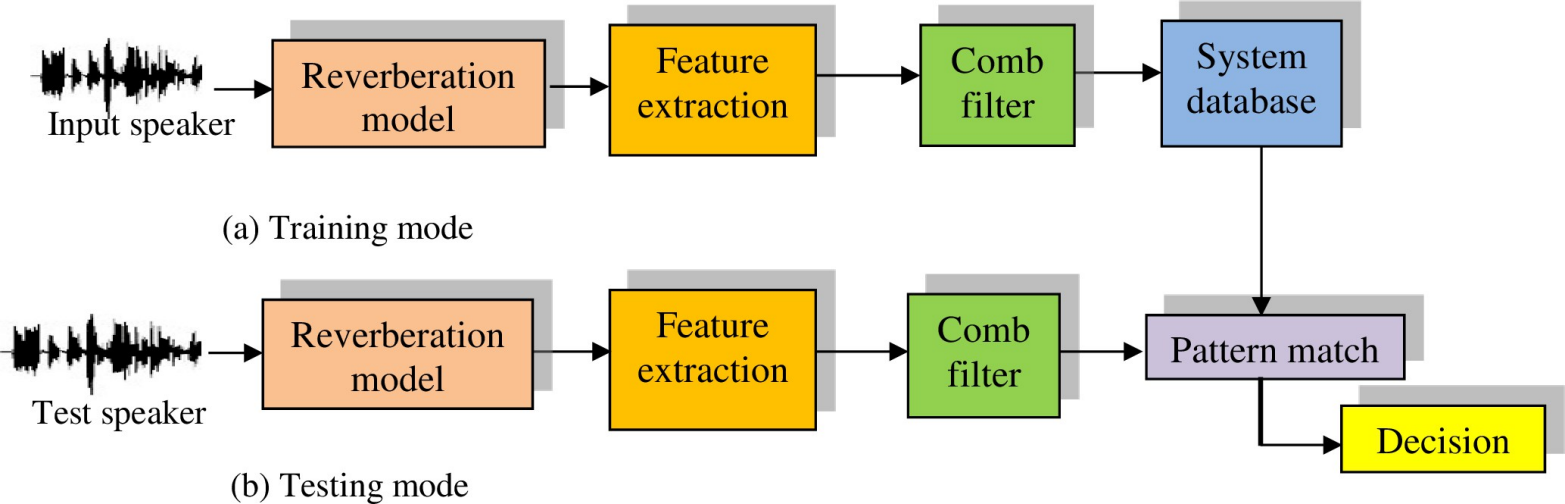
Cancelable speaker identification in the presence of reverberation.

In this sub-section, we present a cancelable speaker identification system on the feature level in the presence of reverberation. The intended degradation is induced with a comb filter model on the feature level in both training and testing modes as shown in Fig 11.

## 8. Simulation results and discussion

### 8.1 Speech database

Initially, a database was assembled, comprising recordings for 15 distinct speakers. Each speaker was tasked with repeating a specific Arabic sentence a total of 10 times. During the training phase, a total of 150 speech samples were employed to derive Mel-Frequency Cepstral Coefficients (MFCCs) and polynomial coefficients, which were subsequently utilized to construct the feature vectors for the database.

In the testing phase, each of the aforementioned speakers was prompted to recite the designated sentence once more, after which their speech signals underwent a degradation process. From these degraded speech signals, comparable features to those utilized during training were extracted. These features were then employed for the matching process.

The features consist of 13 MFCCs and 26 polynomial coefficients, collectively composing feature vectors comprising 39 coefficients for every frame within the speech signal. The speech signals have a sampling frequency of 18,000 samples per second. The speech database is summarized in Table 1.

**Table 1:** Speech database description and ANN parameters.

Parameter	Value
No. of speakers	15 [10 male + 5 female]
Sampling frequency (fs)	18 kHz
Platform	Matlab 2017b and single GPU
Stride	2
Batch size	128
Learning rate	0.001
Reverberation time (*T*_*R*_)	0.5s

This paper delves into the analysis of speech signals in environments marked by indoor noise such as home noise. The source of noise comes from interference from another speaker, or from the surrounding environment, and modeled as Additive White Gaussian Noise (AWGN). In this work, when the speech signal is corrupted with noise, it is processed by means of considered transforms such as the DCT, DST, and DWT.

Various simulation experiments have been executed to rigorously test the proposed systems for speaker identification and cancelable speaker identification. The assessment encompassed diverse feature extraction schemes, including:

1. Features derived directly from speech signals.
2. Features extracted from the DWTs of speech signals.
3. Features obtained from both speech signals and their corresponding DWTs.
4. Features derived from the DCTs of speech signals.
5. Features obtained from both speech signals and their corresponding DCTs.
6. Features originating from the DSTs of speech signals.
7. Features derived from both speech signals and their corresponding DSTs.

**Fig 12:**
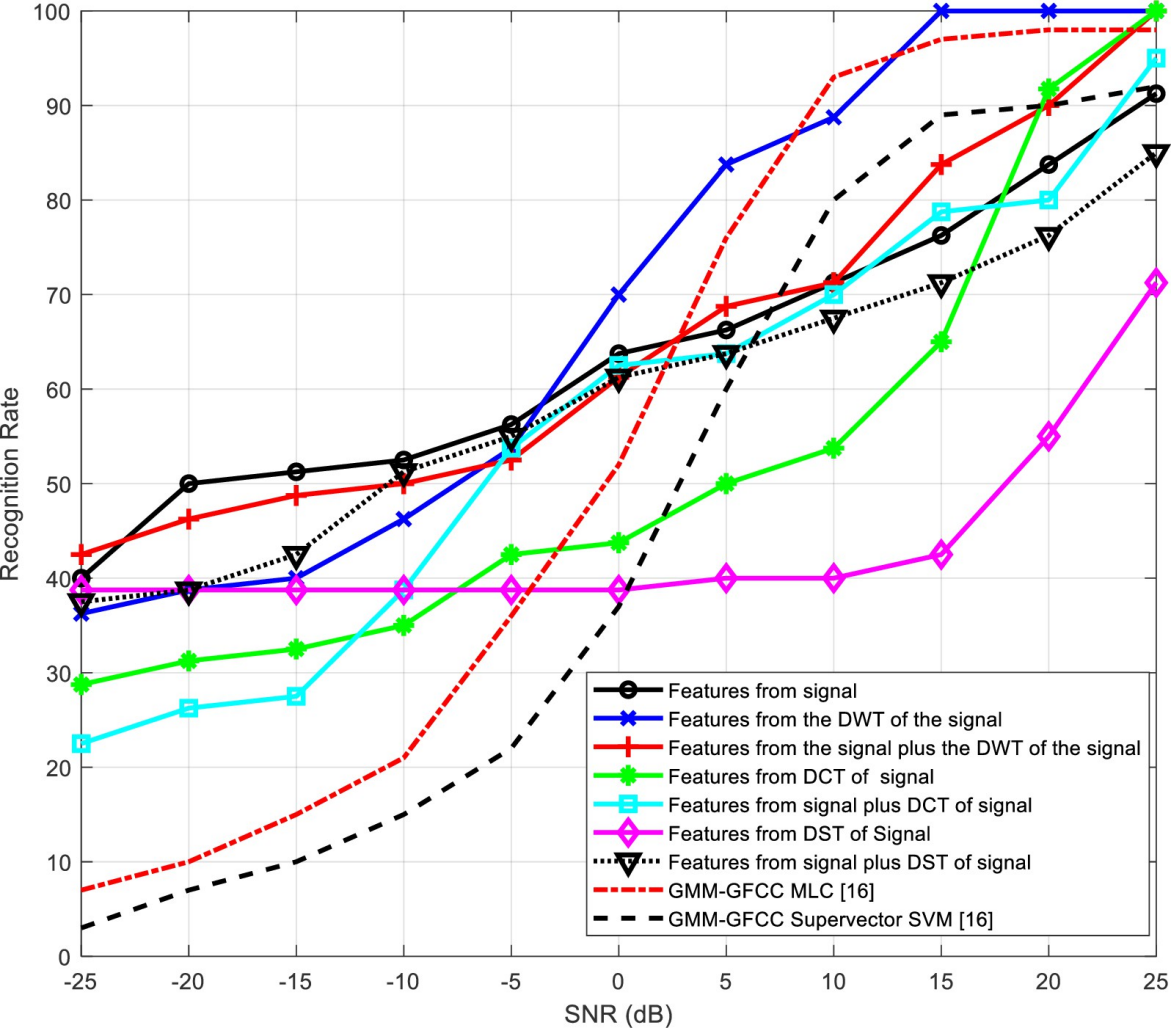
Variation of the output recognition rate of the speaker identification system with SNR for different feature extraction techniques without reverberation effect.

**Fig 13:**
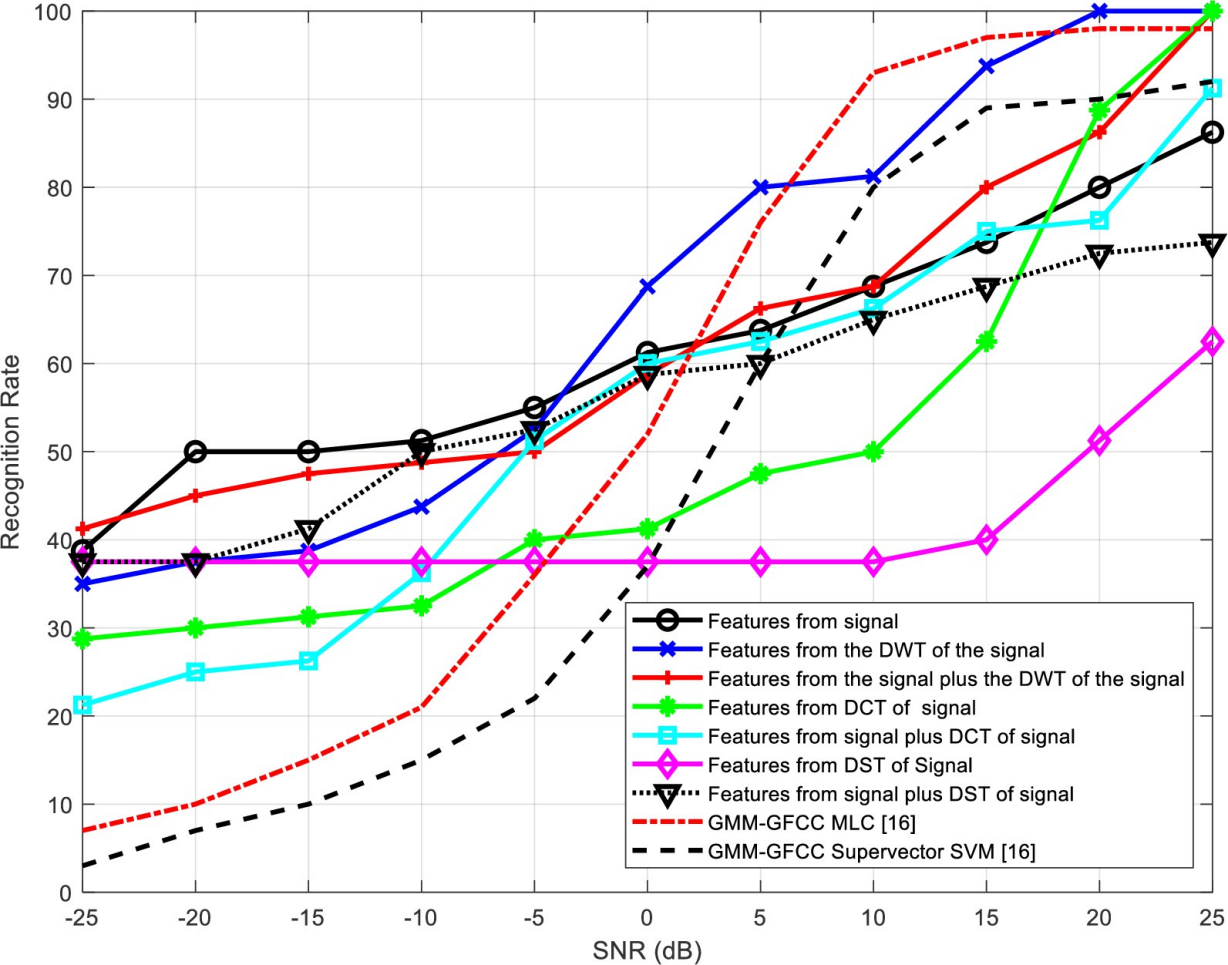
Recognition rate variation in the presence of reverberation, as influenced by diverse feature extraction techniques across different SNR levels.

**Fig 14:**
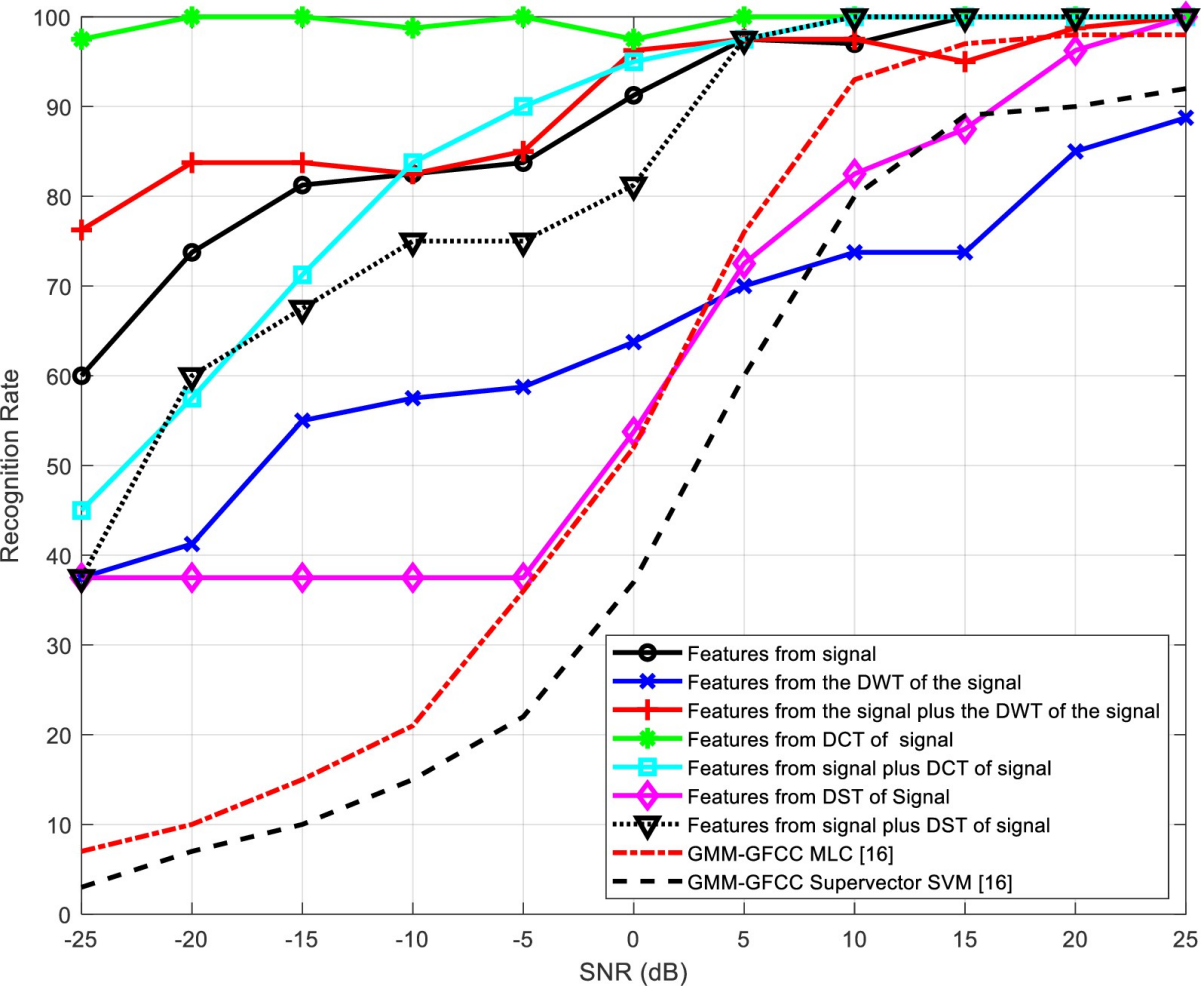
Variation of the output recognition rate of cancelable speaker identification system with SNR for different feature extraction techniques without reverberation effect.

**Fig 15:**
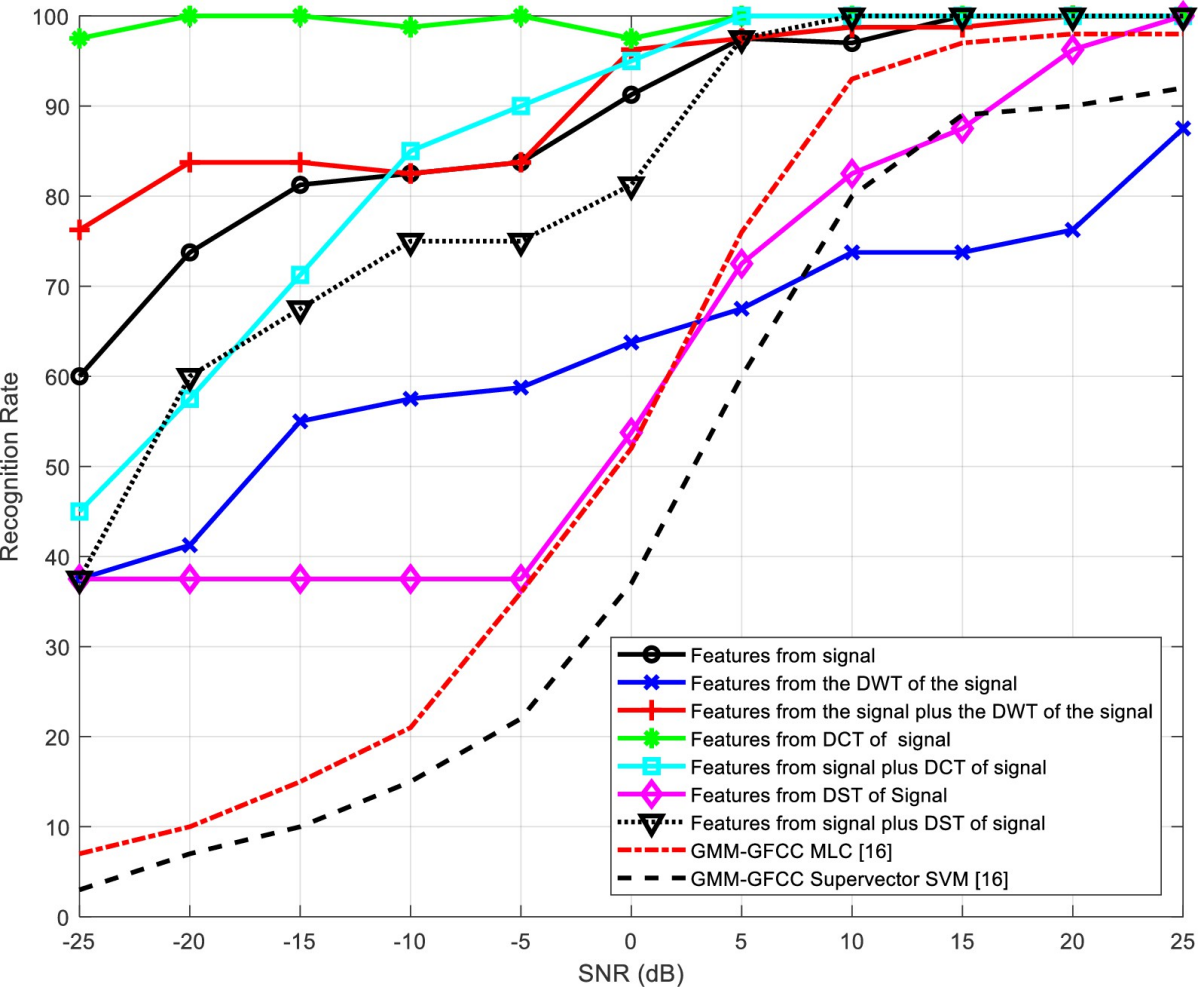
Recognition rate variation in the cancelable speaker identification system under reverberation, across different feature extraction techniques and SNR levels.

Table 2 presents the number of epochs required for training the neural networks for the different feature extraction schemes. The representation of recognition rate versus SNR is visually depicted in Figs 12 to 15 and substantiated with data presented in Tables 3 to 6.

**Table 2:** Number of epochs required for training the ANN.

	Features from speech signals	Features from DWT of signals	Features from signals + DWT of signals	Features from DCT of signals	Features from signals + DCT of signals	Features from DST of signals	Features from signals + DST of signals
No. of epochs	160	95	110	110	130	85	95

**Table 3:** Output recognition rates of the speaker identification system for different feature extraction techniques at different SNRs without reverberation effect.

% of Recognition Rate							
SNR (dB)	Features from speech signals	Features from DWT of signals	Features from signals + DWT of signals	Features from DCT of signals	Features from signals + DCT of signals	Features from DST of signals	Features from signals + DST of signals
**-25**	40	36.25	42.5	28.75	22.5	38.75	37.5
**-20**	50	38.75	46.25	31.25	26.25	38.75	38.75
**-15**	51.25	40	48.75	32.5	27.5	38.75	42.5
**-10**	52.5	46.25	50	35	38.75	38.75	51.25
**-5**	56.25	53.75	52.5	42.5	53.75	38.75	55
**0**	63.75	70	61.25	43.75	62.5	38.75	61.25
**5**	66.25	83.75	68.75	50	63.75	40	63.75
**10**	71.25	88.75	71.25	53.75	70	40	67.5
**15**	76.25	100	83.75	65	78.75	42.5	71.25
**20**	83.75	100	90	91.75	80	55	76.25
**25**	91.25	100	100	100	95	71.25	85

**Table 4:** Output recognition rates of the speaker identification system for different feature extraction techniques at different SNRs in the presence of reverberation.

% of Recognition Rate							
SNR (dB)	Features from speech signals	Features from DWT of signals	Features from signals + DWT of signals	Features from DCT of signals	Features from signals + DCT of signals	Features from DST of signals	Features from signals + DST of signals
**-25**	38.75	35	41.25	28.75	21.25	37.5	37.5
**-20**	50	37.5	45	30	25	37.5	37.5
**-15**	50	38.75	47.5	31.25	26.25	37.5	41.25
**-10**	51.25	43.75	48.75	32.5	36.25	37.5	50
**-5**	55	52.5	50	40	51.25	37.5	52.5
**0**	61.25	68.75	58.75	41.25	60	37.5	58.75
**5**	63.75	80	66.25	47.5	62.5	37.5	60
**10**	68.75	81.25	68.75	50	66.25	37.5	65
**15**	73.75	93.75	80	62.5	75	40	68.75
**20**	80	100	86.25	88.75	76.25	51.25	72.5
**25**	86.25	100	100	100	91.25	62.5	73.75

**Table 5:** Output recognition rates of cancelable speaker identification system for different feature extraction techniques at different SNRs.

% of Recognition Rate							
SNR (dB)	Features from speech signals	Features from DWT of signals	Features from signals + DWT of signals	Features from DCT of signals	Features from signals + DCT of signals	Features from DST of signals	Features from signals + DST of signals
**-25**	60	37.5	76.25	97.5	45	37.5	37.5
**-20**	73.75	41.25	83.75	100	57.5	37.5	60
**-15**	81.25	55	83.75	100	71.25	37.5	67.5
**-10**	82.5	57.5	82.5	98.75	83.75	37.5	75
**-5**	83.75	58.75	85	100	90	37.5	75
**0**	91.25	63.75	96.25	97.5	95	53.75	81.25
**5**	97.5	70	97.5	100	97.5	72.5	97.5
**10**	97	73.75	97.5	100	100	82.5	100
**15**	100	73.75	95	100	100	87.5	100
**20**	100	85	98.75	100	100	96.25	100
**25**	100	88.75	100	100	100	100	100

**Table 6:** Output recognition rates of the cancelable speaker identification system in the presence of reverberation for different feature extraction techniques at different SNRs.

% of Recognition Rate							
SNR (dB)	Features from speech signals	Features from DWT of signals	Features from signals + DWT of signals	Features from DCT of signals	Features from signals + DCT of signals	Features from DST of signals	Features from signals + DST of signals
**-25**	60	37.5	76.25	97.5	45	37.5	37.5
**-20**	73.75	41.25	83.75	100	57.5	37.5	60
**-15**	81.25	55	83.75	100	71.25	37.5	67.5
**-10**	82.5	57.5	82.5	98.75	85	37.5	75
**-5**	83.75	58.75	83.75	100	90	37.5	75
**0**	91.25	63.75	96.25	97.5	95	53.75	81.25
**5**	97.5	67.5	97.5	100	100	72.5	97.5
**10**	97	73.75	98.75	100	100	82.5	100
**15**	100	73.75	98.75	100	100	87.5	100
**20**	100	76.25	100	100	100	96.25	100
**25**	100	87.5	100	100	100	100	100

Figs 12 and 13 illustrate how the recognition rate of the speaker identification system changes with SNR for various feature extraction techniques, excluding and including the impact of reverberation, respectively. The obtained results are compared with the results presented in [16]. Two different approaches are used for comparison depending on GMM-GFCC MLC and GMM-GFCC supervector SVM.

According to the obtained results, it is evident that the performance of all schemes experiences improvement as the SNR increases. Furthermore, the scheme based on wavelet domain consistently delivers the most robust performance. This superiority can be attributed to the innate ability of the wavelet transform to decompose signals into sub-bands, enhancing the system ability to capture essential features. It is clear also that the proposed method outperforms the other approaches [16], specially at low SNR.

Figs 14 and 15 present the variation of the output recognition rate of the cancelable speaker identification system with SNR for different feature extraction techniques without/with reverberation effect, respectively.

Conversely, within the realm of cancelable speaker identification systems presented in Figs 14 and 15, our findings underscore that DCT-based features outshine others in terms of performance. This can be attributed to the remarkable resilience of few selected DCT coefficients to the distortions introduced by the comb filter. This resilience is a result of the energy compaction property intrinsic to DCT. It is evident that the proposed method consistently outperforms the other approaches [16], particularly under low SNR conditions.

As indicated by Table 1, the obtained results were obtained considering a reverberation time (*T*_*R*_) of 0.5 s. The effects of changing reverberation time can be described as follows; longer reverberation times can degrade speech quality and make it more challenging to recognize speakers, accurately. The increased presence of reflections and echoes can introduce additional acoustic variability, leading to a decrease in Recognition Rate (RR). Shorter reverberation times, on the other hand, indicate less reflection and echo in the environment. This can lead to cleaner speech signals, making it easier for speaker recognition systems to operate with higher accuracy, and, thus, potentially result in improved recognition rates.

Tables 3–6 summarize the obtained results presented in Figs 12–15, respectively. The results highlight that all systems exhibit improved performance with SNR increase. Wavelet-domain features consistently outperform other features in speaker identification systems, regardless of the presence of reverberation, owing to their sub-band decomposition capability (Tables 3 and 4). In contrast, in cancelable speaker identification systems (Tables 5 and 6), DCT-based features enhance performance due to the exceptional resilience of specific DCT coefficients to distortions induced by the comb filter, a trait attributed to the DCT inherent energy compaction property.

## 9. Conclusion

This paper has shed valuable light on the performance dynamics of various speaker identification systems, notably in the presence of challenging acoustic factors such as reverberation and noise. It is evident that the SNR plays a pivotal role in influencing the performance of these systems, with higher SNR levels consistently yielding enhanced results. Specifically, our analysis reveals that, within the realm of speaker identification systems, both in the absence and presence of reverberation effects, wavelet-domain features emerge as the top-performing choice. This superiority can be attributed to the inherent sub-band decomposition capabilities offered by the wavelet transform. The decomposition into different frequency scales enables a more robust representation of speech features, making it particularly resilient in challenging acoustic environments. In contrast, for the cancelable speaker identification system, our findings demonstrate that features based on DCT deliver the most favorable performance. This can be attributed to the remarkable ability of a select few DCT coefficients to withstand the distortions introduced by the comb filter, thanks to the energy compaction property inherent to the DCT.

## 10. Future work

Future work can focus on further refining of cancelable speaker identification techniques, potentially exploring advanced signal processing methods and expanding the scope to address emerging challenges in biometric security. The effects of outdoor noise such as car and street noise can also be stuied. Additionally, investigating the adaptability of these systems to real-world scenarios and exploring the integration of emerging technologies, such as deep learning, holds promise for continued advancements in the field. Furthermore, exploring the integration of cutting-edge technologies, particularly deep learning, offers a promising avenue for further advancements. Deep learning models, with their capacity for feature extraction and pattern recognition, can potentially revolutionize speaker identification systems, enhancing accuracy and robustness.

Moreover, as the landscape of biometric security evolves, future work should address emerging challenges, such as adversarial attacks and multimodal authentication, to ensure comprehensive protection against evolving threats. Collaborative research efforts and interdisciplinary approaches could also unlock novel avenues, encompassing fields like acoustic forensics and human-computer interaction.
