# Expression of Concern: Enhancing speaker identification through reverberation modeling and cancelable techniques using ANNs

**DOI:** 10.1371/journal.pone.0349268

**Published:** 2026-05-13

**Authors:** The PLOS One Editors

After this article [1] was published, the following concerns were noted:

- The Data Availability statement refers to speech data from a Chinese Mandarin Corpus, however Section 8.1 reports on a dataset of Arabic speech samples. The source and demographics of this cohort are insufficiently described in the article, and the underlying dataset was not provided.
- The *PLOS One* Editors identified concerns about authorship.

The corresponding author has provided demographic information for the sample cohort, as well as the collection protocol.

They stated that the demographics and collection protocol for the Arabic speech sample dataset described in Section 8.1 are as follows: 15 adult volunteers (9 male, 6 female) that were native Arabic speakers aged 22–45 years with no known speech or hearing impairments. Speech samples were recorded in 2023 in a controlled acoustic environment at Menoufia University, using a 16 kHz sampling rate and 16-bit resolution. The authors did not provide the dataset or comment on its availability.

The corresponding author’s responses did not resolve the authorship concerns.

In light of the concerns, the *PLOS One* Editors issue this Expression of Concern. Readers are advised to interpret the article [1] with caution.
